# What Does Addiction Mean To Me

**DOI:** 10.4103/0973-1229.27609

**Published:** 2006

**Authors:** Morten Hesse

**Affiliations:** * *Aarhus University Centre for Alcohol and Drug Research Købmagergade 26E DK-1150 Copenhagen. Email : hesse@medscape.com*

**Keywords:** *Addiction*, *Dependence*, *Causes and Consequences of Addiction*

## Abstract

Addiction is compulsive need for and use of a habit-forming substance. It is accepted as a mental illness in the diagnostic nomenclature and results in substantial health, social and economic problems. In the diagnostic nomenclature, addiction was originally included in the personality disorders along with other behaviours considered deviant. But it is now considered a clinical syndrome. Addiction is multifactorially determined, with substantial genetic influence. The development of addictions is also influenced by environmental factors, and an interplay between the two. In the clinical context, addiction puts problem substance use on the agenda, and helps focus on the difficulties associated with drug use. But the concept of addiction is also used to distance the user from addicts, and in this way, may be counter-therapeutic. The addiction concept has also had a substantial influence on policy. The almost universal prohibition against drugs such as opiates, cocaine, cannabis and amphetamine has much support. But unfortunately, it has not been able to hinder the development of substance use problems. Optimism is fostered by the development of respectful ways of thinking about people with addictions, in particular, from advocates of motivational interviewing.

## Introduction

Addiction is a term that means compulsive physiological need for and use of a habit-forming substance (like heroin or nicotine), characterized by tolerance and well-defined physiological symptoms upon withdrawal; it has also been used more broadly to refer to compulsive use of a substance known by the user to be physically, psychologically, or socially harmful (Maddux and Desmond, 2000). In the following essay, I shall try to explore the meaning of this concept.

A note on the term addiction: I shall use the term *addiction*, except when I refer to a specific diagnostic system that uses other terms. The term *dependence* was introduced to reduce the stigma associated with addiction in the 50ties. I generally prefer the term *addiction*, because the term *dependence* is sometimes used in a counter-therapeutic fashion: I *depend* on my heroin/cigarettes etc. *Addiction* more directly addresses the issue: I am *addicted* to heroin/cigarettes, which means that I suffer various consequences from using the drug, and I will need to think about whether I will accept these consequences, or do something about my addiction (Maddux and Desmond, 2000).

## What Are Addictions?

Addictions are disorders related to use of substances. Substances in this context include intoxicants, such as alcohol or heroin, or milder stimulants, such as caffeine or nicotine. Addictions include dependence and other drug use disorder listed under psychiatric disorders in both the World Health Organisation's International Classification of Diseases, 10th revision [ICD-10] and the American Psychiatric Association's [APA] Diagnostic and Statistical Manual, 4th revision [DSM-IV] (American Psychiatric Association 2000; Maddux and Desmond, 2000; Nathan, 1991).

Addiction is a form of mental illness, and as any mental illness, it is associated with stigma. Mental illness is a broad generic label for a category of illnesses that may include affective or emotional problems, behavioural dysregulation, and cognitive dysfunction or impairment. Most evident in addiction is behavioural dysregulation: addiction is, by definition, problems controlling consumptive behaviour. Emotional instability and cognitive dysfunctions may or may not accompany the disorder.

## The Impact Of Drug And Alcohol Addiction

A negative impact of some drugs, including drugs such as alcohol and opiates, has long been acknowledged. For instance, Commissioner Lin of the Chinese Empire wrote to Queen Victoria of the British Empire, urging her to take action against opium export. Lin stressed the negative consequences of opium use on Chinese society (Lin, 1839). The Quran, the holy book of Islam, written in the early 7th century, states:

Satan's plan is (but) to excite enmity and hatred between you, with intoxicants and gambling, and hinder you from the remembrance of Allah, and from prayer… [Quran, 5.91; Razwy (ed.,) 2000].

In England, in 1436, Bishop Chandler warned the monks at the Monastery of Abbotsbury that, “…wine and women cause men to err…” (Page, 1908).

However, condemnation of intoxicants is far from universal. Even when substance use causes problems, some observers suggest temperance, others abstinence, yet others prohibition. But the observation that use of intoxicants cause harm to society is common across time and place.

## Health Consequences

Addictions, or substance use disorders, are prevalent and affect public health in a number of adverse ways. The World Health Organization has estimated that alcohol, tobacco and illicit drugs combined are responsible for 12.5% of all deaths worldwide (see online links: http://www.who.int/substance_abuse/publications/en/global_status_report_2004_overview.pdf and http://www.who.int/substance_abuse/facts/global_burden/en). Specifically, alcohol use is responsible for 8.8% of annual deaths, tobacco for 3.2%, and illicit drugs for 0.4% (World Health Report, 2002).

Many diseases, including cardiovascular disease, stroke, cancer, HIV/AIDS, hepatitis, respiratory disease, psychosis, depression and anxiety can be affected by substance use. Some of these effects occur when drugs are used at high doses or after prolonged use, but some may occur after just one use. The health status of drug abusers is generally affected by their drug use, and life expectancy of drug misusers is often much lower than that of the general population.

## Social Consequences

Addiction affects not only substance users. It also affects other than those who consume substances directly. For example, children are affected by their parents’ drinking. In young children living with substance abusers these effects can be observed, and drug abuse causes even worse problems than alcohol use (Cooke, Kelley, Fals-Stewart and Golden, 2004). High density of alcohol outlets, both bars and off-premise sales, have been associated with a number of adverse social and health outcomes, such as gonorrhoea, violence, motor vehicle accidents, and child abuse and neglect. Illicit drug users are often exposed to accidents, and are very often victims of violence.

There are even socio-economic negative consequences of substance addiction. People with alcohol or drug addiction are less likely to be working, and alcohol addiction is associated with leaving the workforce prematurely (Romelsjo, Stenbacka, Lundberg and Upmark, 2004). Drug abusers cost large sums in hospital beds, as do alcohol abusers. Imprisonment, health costs, and loss of productivity contribute to the costs of alcohol and drug addiction (Fenoglio, Parel and Kopp, 2003). Unlike other psychiatric groups, substance abusers are also more likely than community controls matched on age and gender to commit crimes, including violent crimes.

The causal nature of problems associated with substance use is difficult to entangle precisely. Patterns of use and total amount of use may be differentially related to problems (Peele, 1997). For instance, total amount of use is related to health consequences and mortality in the general population, but binge use pattern is associated with violence and interpersonal consequences. Also, in some countries, the link between alcohol and aggressive behaviour, such as homicide, is stronger than in other countries (Rossow, 2001).

In order to show a causal link between addiction and personal and social consequences, it is necessary to show that reducing substance use reduces adverse effects of drug use on society or other individuals. It is plausible that some of the problems associated with substance addiction are linked because of common underlying causes. For instance, a person who is unable to cope with any kind of cooperation or interaction with other people may develop alcohol addiction. Does the addiction cause the unemployment of this person? Or would he be unemployed regardless of addiction, because sober or intoxicated, this person is just not able to function in a workplace? With these reservations, the evidence is sufficient to say that substance use negatively affects society and many people in society.

## The Historical Development Of Substance Use Disorder Diagnoses

Before emergence of the official diagnostic nomenclature, a range of concepts were coined to describe the problematic use of substances: terms such as addiction, inebriety, intemperance, alcoholism, euphomania, and others (Maddux and Desmond, 2000). Many of them reflecting a view of addiction as a moral deficiency. Later, it was suggested that substance use disorders be subclassified into a subtype with physical dependence (“addiction”) and a subtype without dependence (“habituation”) (Maddux and Desmond, 2000).

In 1952 with the first Diagnostic and Statistical Manual [DSM-I], only one classification was possible for drugs (drug addiction) and one for alcohol (alcohol addiction). These were listed under sociopathic personality deviance, a section that also contained antisocial behaviour and deviant sexuality. These behaviours were assumed to be dangerous to society, and generally, people who exhibited such behaviours were thought incapable of changing. The ICD-7, published in 1957, also used the term *drug addiction*, and subclassified alcoholism into three subcategories: chronic, acute and unspecified.

Classification of substance use disorders remained the same in DSM-II. In the 50ties, addiction was recoined into “dependence” by the World Health Organization, and in 1980 APA followed suit in the DSM-III (Maddux and Desmond, 2000). With the DSM-III in 1980, substance use disorders were subclassified into *dependence* and *abuse*. This division of substance use disorders into two sub-categories reflected different changes in the view of addictions. The study of tolerance and withdrawal in drug addiction had gained increasing interest, leading to a need for a sub-category of drug misuse characterized by the presence of these phenomena. Also, research had shown that not all drug or alcohol problems showed the chronic course that was assumed in the classification of substance use disorders along with sociopathic deviance.

Finally, the psychiatric community wanted to remove the stigma from alcohol dependence that came from being classified along with deviant sexuality and antisocial behaviour. With the DSM-III-R in 1987, specific criteria were formulated for many psychiatric disorders, including substance use disorders. These criteria were a mixture of physiological changes associated with substance use, such as tolerance and withdrawal, and more psychological phenomena such as loss of control and priority of substance use over other activities. These criteria have undergone little change with the DSM-IV in 1994, except that the diagnostic criteria became somewhat more restrictive, while the alcohol abuse criteria became less restrictive.

## The Current Diagnoses For The Addictions

### The Diagnostic And Statistical Manual, 4th Revision (DSM-IV)

According to the DSM-IV, two kinds of addiction can be diagnosed: substance abuse and substance dependence (American Psychiatric Association, 2000). *Abuse* is a pattern of alcohol use, leading to social, occupational, or medical impairment. A diagnosis of abuse can only be given in the absence of a diagnosis of dependence.

A diagnosis of alcohol *dependence* requires at least three or more of the following: (1) Tolerance (2) Withdrawal symptoms, or drinking to avoid symptoms (3) Loss of control (4) A persistent desire or efforts to cut down or control drinking (5) A great deal of time spent drinking, or recovering from drinking (6) Social, occupational, or recreational activities given up because of alcohol use (7) Drinking continues despite having a persistent or recurrent physical or psychological problem that is likely to have been worsened by alcohol.

The withdrawal symptoms can differ by substance taken. Some substances, such as cannabis or hallucinogens, do not produce a characteristic withdrawal syndrome, and withdrawal is not listed as a criterion for these substances.

### The International Classification Of Diseases, 10th Revision (ICD-10)

The International Classification of Diseases, 10th revision [ICD-10] includes diagnoses of harmful use for alcohol or substance dependence, for any substance, including alcohol. Harmful alcohol use is defined as heavy alcohol use (quantity defined by local standards, e.g. over 28 drinks per week for men, over 14 drinks per week in woman), and that overuse of alcohol has caused physical harm (e.g. liver disease), psychological harm (e.g., depression) or has led to harmful social consequences (e.g., unemployment).

Substance dependence is defined by either current use or a current persistent and strong desire to use the substance, plus two or more of the following: continued substance use despite harm, difficulty controlling use, tolerance, and withdrawal.

## Similarities And Differences Between DSM-IV And ICD-10

The criteria are summarized in Table 1. DSM-IV has more optional criteria for both dependence and abuse than ICD-10. In the DSM-IV, 3 of 7 criteria are needed, whereas in the ICD-10, 3 of 5 criteria are needed, including the obligatory criterion of use or desire to use. Therefore, the DSM-IV diagnosis is less restrictive, resulting in higher prevalences for DSM vs. ICD in studies where both set of criteria have been applied to the same populations (Nurnberger *et al*., 2004). The milder abuse/harmful use diagnoses have no overlapping criteria, and are clearly not the same thing. Therefore, agreement between the two dependence diagnoses is generally better than between the harmful use and abuse diagnoses (see, for example, Giang, Spak, Dzung and Allebeck, 2005).

**Table 1:** Criteria for Addiction Diagnoses in the DSM-IV and ICD-10

	DSM-IV	ICD-10

**Dependence**	**3 or more of…**	**3 or more of…**
Tolerance	*	*
Withdrawal	*	*
Loss of control	*	*
Unsuccessful attempts to cut down	*	
Spending much time	*	
Reduction in other activities	*	
Continued in spite of problems	*	*
Desire to use the substance or ongoing use		R
Substance abuse	R	
**Abuse/Harmful use**	**1 or more of…**	**Both…**
Interferes with role obligations	*	
Hazardous use	*	
Legal consequences	*	
Use despite interpersonal problems	*	
Use more than general population		R
Negative health consequences		R

**Note:** Criteria marked with an asterix [*] are optional, but criteria marked with R are required.

There are also differences in content in the dependence diagnoses. The DSM-IV has an optional criterion of unsuccessful attempts to quit. The ICD-10 requires in a sense the opposite, namely, either use or desire to use. The desire to quit as a criterion suggests a more cooperative patient than the desire to use when not using. It is a paradox in the DSM-IV that the heavy user who wants to deal with his problem is considered more ill than the heavy user who does not want to deal with the problem, all other things being equal. In favour of the DSM-IV criterion, unsuccessful quit attempts point in the direction of loss of control. And a case can be made that regardless of the amount of drug or alcohol use, no diagnosis of dependence should be made if a person is able to quit at will.

## Causes And Mechanisms Of The Addictions

Addictions arise when substance use becomes disordered, when substances are used more and more in situations where they do harm, and when the user loses control over the use. But how and why does substance use become disordered? Is there individual variability in our vulnerability to develop addictions?

### 1. Heritability

Recent research on addiction has added to our understanding of factors that contribute to the development of addictions, including genetic risk factors (Goldman, Oroszi and Ducci, 2005). How can addiction be genetically determined? According to the Internet encyclopaedia, Wikipedia (2006):

Genes are regions of nucleic acid that parents pass to offspring during reproduction as chromosomes in nuclei of gametes. These entities encode information essential for the construction and regulation of proteins (such as enzymes) and other molecules that determine the growth and functioning of the organism.

But consumption of drugs is neither protein, nor enzyme. It is not a physiological process that can be determined by the presence or absence of a protein or an enzyme. It is not a psychological process that can be assumed to be determined by the presence or absence of a protein or an enzyme. It is a behaviour that relates to a substance outside of the organism.

The process from gene to addiction is mediated by physiological, psychological, and social processes. In order for genes to increase the risk of addiction, genes must enhance physiological processes that in turn increase the likelihood of psychological processes that in turn increase the likelihood that once drugs are encountered, they will also be consumed, and/or that once drugs are consumed, the person will continue to consume them. Therefore, in addiction as in psychiatry in general, there are no “genes for X disease”.

Addiction has moderate to high heritability, higher than most psychiatric disorders, and this heritability is determined by multiple genes that both interact and work independently of each other. There is also evidence that the use of drugs is partly determined by genetic factors, although the heritability of drug use is weaker than the heritability of drug addiction. In terms of drug use, the genetically predisposed is more likely to use intoxicants in general, rather than specific substances.

Drugs that tend to produce addiction easily, such as cocaine, opiates and tobacco smoking, also show the greatest heritability. Drugs that are less likely to cause addiction, such as cannabis, have the weakest heritability coefficients. This pattern of results is as expected, since the mechanisms that underlie addiction are themselves inheritable.

The impact of multiple independent genes means that humans are born with a degree of risk, rather than with absence or presence of disposition for the disorder.

Addiction liability is not inherited in the same way as, for instance, cystic fibrosis is. In the case of cystic fibrosis, one is either born without any risk, or with risk of the disease, depending on the presence or absence of a single gene. Inheritance is much in the same way that height or intelligence is inherited: people are not born with either a gene variant that predisposes them to become 140 centimeters, or with a gene variant that predisposes them to become 190 centimeters. Rather, they are born with a combination of multiple genes that predispose them to grow to some degree of height, given sufficient nutrition is available.

A different consequence of the fact that multiple genes determine addiction risk is that genetic risk for addiction may both be a risk factor for developing a specific addiction (for example, alcohol), and be a risk factor for addiction to two or more substances. Let's take an instance. Alcoholism and nicotine addiction, for example, are both comorbid and cross-transmitted. Approximately 85% of people with severe alcohol addiction smoke. Around 50% of the genetic liability to nicotine dependence is shared with alcoholism, and 15% of the genetic liability to alcoholism is shared with nicotine dependence (Swan, Carmelli and Cardon, 1997). Thus, the increased genetic risk for alcohol addiction is small, although it exists, given the liability for tobacco addiction. But the increased genetic risk for tobacco dependence is large given a liability for alcohol addiction.

Multiple independent genes also means multiple pathways, which probably lead from drug genetic disposition to drug addiction. For instance, genetic disposition for stress sensitivity, impulsivity and externalizing behaviour, sensation seeking, and proneness to anxiety and dysphoria may all contribute to the development of drug addiction.

Therefore, it is not surprising that alcohol dependence is associated with familial aggregation of a range of psychiatric conditions, including depression, antisocial personality disorder, dysthymia, general anxiety, obsessive-compulsive disorder, post-traumatic stress disorder, and addiction to several other substances (Nurnberger *et al*, 2004).

### 2. Environmental Risk Factors

There is little doubt that there also are environmental risk factors for the development of addictions. Obviously, substances must be accessible for addiction to develop. Reducing access to alcohol reduces alcohol-related problems. Similar observations have been made for heroin. When drugs are available, people must use them in order to develop addiction. There is evidence that early exposure leads to increased risk of developing addiction. A widely believed model states that some drugs function as *gateway-drugs*, and that there is a general, or even natural, progression from one type of drugs to another. Some drugs (e.g., alcohol) act as gateways, and youth who do not start drinking alcohol rarely progress to marijuana; similarly, almost no cocaine users have not used marijuana previously. This model does not predict that the majority of users must necessarily proceed to the next drug in the sequence, but only that use of drugs later in the sequence is unlikely in the absence of use of drugs earlier in the sequence (Kandel and Yamaguchi, 1993).

Whether use of *gateway drugs* is an environmental risk factor or an indicator of addiction liability is an important question. There are arguments for both: any addictive drug can alter brain functioning in ways that increase vulnerability to addiction, which makes it probable that substance use is an environmental factor that increases risk of future addictions (Robinson and Berridge, 2000). But the common heritability of addiction to various drugs suggests that common genetic factors can underlie tobacco, alcohol, marijuana and heroin use (Goldman *et al*, 2005). The empirical question is how much actual causation from use of gateway drugs to use of the next drugs is possible, after genetic vulnerability is accounted for.

Beyond availability and use, there is some evidence that environmental stress increases the risk of relapse in patients with addiction (Brown, Vik, Patterson, Grant and Schuckit, 1995), although the specific pattern of stressors that predict relapse may differ by psychiatric co-morbidity (Tate, Brown, Unrod and Ramo, 2004).

The evidence for the stress-substance use link in rats suggests that the relationship is very complex, and depends on a number of factors. The effect of stress on behaviour in rat models is stressor-specific, and to some degree, procedure- and drug-class-specific (Lu, Shepard, Scott Hall and Shaham, 2003). Psychosocial risk factors also seem to influence cocaine use in non-clinical samples, although it is subjective stress more than objective stressors that are associated with use (Karlsgodt, Lukas and Elman, 2003).

Generally, and independent of socio-economic status, lack of coping skills appears to be more important for problematic substance use and relapse than adverse life events (Miller, Westerberg, Harris and Tonigan, 1996).

It is well established that in some socio-economic strata, substance use disorders are more common than in others. Socio-economic factors are related to substance use in a complex way. For instance, American college undergraduates in the 70ties and 80ties who used drugs differed little from non-users on academic performance, and were as active as non-users in extra-curricular activity at their university. In 1999, drug users were fewer than in earlier years, and they differed much more from non-users, both by performing more poorly academically and by not being involved in college activities (both studies reported in Pope, Ionescu-Pioggia and Pope, 2001). Thus, within the same socio-economic strata, it appears that in different time-periods, different factors are associated with substance use.

### 3. Learning And The Environment

The complexity of environmental influences on drug and alcohol use suggests that cultural and psychological mechanisms must be taken into account. Cultural and psychological mechanisms have been suggested by symbolic interactionism and social learning theory. In a classical article, *Becoming a marijuana user*, Howard Becker (1953) presented a model for learning the ‘correct’ way to interpret the effects of cannabis through social interaction with other users. His point of view was that cannabis did not give euphoria until the user learns to recognize the effects and connect them with drug use; and learns to enjoy the sensations he perceives.

Several developments have been made in this area of theory, and Becker's radical view is now generally considered obsolete. Social learning theory has suggested several potentially important mechanisms (Marlatt and Gordon, 1984). For instance, expectations about drug effects can have an impact on both the effects of drug use and consumptive behaviour (Donovan and Marlatt, 1980). The environmental factors that influence substance use in humans are in summary more cultural than socio-economic. The right to drink alcohol has in Western societies been a symbol of independence and power that has been associated with the Caucasian adult male (Valverde, 1998). It is the symbolic meaning of substance use and its variations across cultures, times and groups that defines the environmental influences on substance use in humans (Valverde, 1998).

## The Clinical Perspective – Addiction As A Language Of Communication About Substance Use Problems

In the clinical context, addiction is a concept that helps professionals and patients acknowledge that substance use is a source of problems. When professionals receive training in addiction, they increase their awareness that use of substances may cause patients problems. When patients learn about addiction, they may become aware of the fact that substance use may harm them. This, more than anything, is the significance of the addiction concept in the clinical context.

When professionals, such as doctors, successfully screen patients for substance use problems, the result is not so often a comprehensive treatment plan, as simply awareness raising on the side of the patient. For instance, the patient may complain of stomach problems, the doctor may diagnose an irritable bowel syndrome, worsened by a vicious circle of alcohol drinking and stress. After uncovering this vicious circle together with his physician, the patient may begin to consider cutting down on his drinking.

Some patients dislike the idea of being addicted to something. For instance, some smokers or drinkers quit because they do not like the idea of being controlled by a chemical substance. Thus, acknowledging addiction may be an important step towards changing the behaviour. As always in addiction treatment, and other psychiatric therapies, the personal values of patients are important in treatment. For instance, the social worker who works with addicts may become motivated to quit smoking after having realized that his cigarettes are in fact “fixes”. The father who sees himself as a role model for his son may become motivated to cut down on his drinking after seeing his 3-year old son playing drunk.

## The Other Side Of The Addiction Concept

Putting a theme on the agenda is not the same as successfully resolving the problem. The addiction concept can be used for other purposes than simple awareness raising. For instance, patients may use the addiction concept to distance their own use of a substance from others’ use, and therefore in fact make light of their use.

### 1. Adverse Effects Of The Addiction Concept On Clinical Practice

Patients who receive advice to reduce their drinking may distance themselves from “true alcoholics” who are “on the skid row”. In this way, stigma associated with addictions may be counter-therapeutic in their treatment.

Even among patients on methadone maintenance treatment, or in inpatient treatment services, it is not uncommon to come across patients who distance themselves from “true addicts”, “alcoholics”, or “junkies who use needles”. This use of the addiction label leads the patient to view his substance use as relatively unproblematic; and he, implicitly or explicitly, questions whether anything should be done about his substance use at all. Very often such patients end up relapsing after treatment.

As pointed out by Miller and Rollnick (1991), this mechanism often emerges in the interaction between patient and physician, or other professionals. The professional may try to confront, even argue, that the patient has a problem, and the patient withdraws from the discussion. The eager professional may return to the subject of substance use, knowing that the substance use causes problems, and thereby make the patient even more defensive.

Also, patients may argue that “dependence” or “addiction” means that they cannot change. They may argue that although it is clear smoking is already damaging their health (or that alcohol is damaging their family), they cannot change, because, “I am dependent, I am unable to quit – sorry”. Working through such defences and faulty coping mechanisms may make all the difference between therapeutic success and failure.

### 2. Adverse Effects On Prevention

The use of the addiction concept to make light of the problem is not restricted to users. A focus on addiction and dependence liability may derail the discussion on benzodiazepine use, for example, because the question is not whether an elderly patient will develop “addiction”, but whether the medication is causing unacceptable risks. For instance, elderly patients or nursing home residents, who take benzodiazepines or drink alcohol, may risk serious injuries. Discussing whether elderly people will develop “addiction” is not important, and may remove focus from the more salient risk of falls and psychological side effects.

Similarly, if universal or selective prevention is aimed at to reduce the risk for dependence, other equally important goals of prevention may be missed. For instance, youth who start smoking cannabis may develop cannabis addiction, but most do not. However, a far more salient risk for cannabis smokers is the development of psychosis (Smit, Bolier and Cuijpers, 2004). Youth who drink alcohol excessively may be at risk of becoming alcohol dependent. But few actually do. However, excessive drinking may cause a number of other problems, including accidents, unwanted sex, and violence.

A focus on addiction may weaken the impact of universal prevention programmes, because very few, even among patients who are actually at risk of developing dependence, will themselves acknowledge alcohol or other substance use as “dependent use”. Few youth who are at risk of getting involved in drugs believe police officers or teachers who tell them that using cannabis will lead them to become homeless street addicts. Adults who drink too much are unlikely to identify with “alcoholism” or “alcohol dependence”, but may experience morning fatigue, putting on weight, irritability and similar side effects from their drinking and may identify with those problems.

## Addiction And Policy

Addiction is a concept that has had substantial influence on policy, perhaps more so than any other mental illness. In many countries, substances such as alcohol and tobacco are restricted to adults, and are taxed and can only be sold from authorized outlets. In almost all countries in the world, substances such as cannabis, opiates, cocaine, amphetamine and many others are considered illegal. While it is argued that prohibition reduces the availability of these drugs, and that prohibition is therefore in effect universal prevention, there are also those who question the effectiveness of the policy, and point to its adverse effects. For instance, African-American communities in the USA have to bear the extra burden of dealing with a large number of men and women released from prison after long sentences for possession of small amounts of marijuana (Iguchi *et al,* 2002).

Human rights organizations, such as Amnesty International, are beginning to question the wisdom of the drug policy because of its negative impact on human rights in the USA (Amnesty International, USA, 2006. See http://web.amnesty.org/library/index/ENGAMR510571998), and in other countries, such as Thailand (Amnesty International, Thailand, 2006. See http://web.amnesty.org/wire/May2003/Thailand). In the USA, but even more so in many poor countries, enforcing the drug policy is coming increasingly in conflict with human rights and its activists. At the same time, very strict drug laws in countries such as Sweden and the USA have not lead to reduction in drug problems. Since the 80ties, successive governments in the USA have passed a number of laws that allow for ever longer prison sentences, allowing police investigators ever more freedom to use what ever methods they deem fit.

However, the war on drugs has become a war on drug users. And that is not the only problem with the war on drugs.

It is, sadly, inefficient for all it costs. When drug manufacturers are cut off from the source of a chemical needed for drug production, they quickly find another to replace it; and although cutting off a legal source reduces problems associated with the drug, the problems quickly return to baseline levels (Cunningham and Liu, 2005). When I hold lectures in small towns all over Denmark, I always ask them whether youth in their town can get hold of drugs easily, and I always get the same answer: yes, they can easily get drugs such as amphetamine and cannabis. My personal view is that states should regulate sales of most substances used for intoxication, including alcohol, cannabis, amphetamine, opiates, cocaine and hallucinogens. States should regulate the price, potency and packaging of these drugs with the aim of reducing harm and reducing consumption.

## Challenges And Reasons For Optimism?

To work with respect and humility is no easy task for the professional, and remains a constant challenge. People who suffer from addictions put themselves, and often also others, at risk through their behaviour. Some are challenging, even aggressive and manipulative (as are many other people with other psychiatric disorders). In some treatment modalities, such as methadone maintenance, patients are given a substance that is illegal, highly toxic at doses that are only slightly higher than the therapeutic doses, and worth a good deal of money when sold illegally. Therefore, it is not surprising that many professionals are suspicious, or even at times hostile, towards some patients. One of the most encouraging developments that I have seen in my work with addictions has been the increasing acknowledgement of the patient as a human being with human rights, who deserves a respectful treatment. In this context, the work of William R. Miller and Stephen Rollnick on “motivational interviewing” is central (Miller and Rollnick, 1991), as is the work of harm reduction advocates, not the least that of G. Alan Marlatt (Marlatt, 1996).

Motivational interviewing is a client-centred, directive method for enhancing intrinsic motivation to change by exploring and resolving ambivalence (Miller and Rollnick, 1991). A number of studies have shown that motivational interviewing is effective in reducing substance use (Burke, Arkowitz and Menchola, 2002). What Miller and Rollnick have taught us, above anything else, is that most people with addictions are ambivalent, and that a patient needs help to acknowledge his ambivalence, rather than “overcoming resistance” or “breaking denial”. What harm reduction advocates have taught us is that even if patients may not accept our goals as professionals, there may still be a great deal that can be done to help the patient avoid suffering and untimely death.

The development towards a more respectful view of people suffering from addictions is not restricted to low-threshold harm reduction or brief intervention. In therapeutic communities, there is a strong trend towards moving away from aggressive confrontation, and treatment models that have traditionally relied heavily on concepts such as “denial” are incorporating principles of respectful communication (Crits-Christoph *et al*, 1999).

When professionals and organizations that provide treatment choose to focus on respectful treatment, they stand a chance of making a difference. When they realize that conflicts can and do emerge, and that some patients are aggressive and manipulative, they stand a chance of dealing with conflicts without loosing respect of the patients. This includes taking up the necessary conflicts and choosing which conflicts are important to take up.

This development cannot and should not mean that we shall focus any less on abstinence from intoxicants for the patients who are interested in such a goal. Indeed, I have met many patients – and people who were not patients – who have benefitted greatly from choosing to abstain from all use of psychoactive drugs.

When patients choose abstinence, they often gain tremendous progress in both their personal development and the social problems associated with substance use. Many of these patients manage to break vicious circles, get a new outlook of themselves and others, and establish new and healthier relationships. And this is what lies at the heart of recovery: not just abstaining, not just stopping to misbehave, but to grow, work and love. If patients do not achieve this goal in overcoming addiction, they may remain as much at risk of an early death as if they had not been abstinent at all (Fridell and Hesse, 2006).

## Concluding Comments

- Addiction is a condition that influences the life of millions of people. Addiction to tobacco and alcohol constitute a serious burden of disease in many societies, and addiction to drugs such as cannabis, amphetamine, heroin and cocaine is associated with a range of negative outcomes.
- Addiction is caused by genetic and environmental factors.
- In practice, addiction can be a helpful concept that allows the public, clinicians and patients to put into words the problems caused by drinking and drug use. However, addiction as a practical concept may have unintended consequences as well, in particular when it allows users of tobacco, drugs and alcohol to distance themselves from “addicts” and thereby make light of the negative consequences of these substances.
- Another adverse consequence of the addiction concept is the “war on drugs” that has been declared decades ago. The addiction concept is used to argue for prohibitions that are inefficient and lead to harm both in drug users and communities themselves.
- By accepting patients’ goals and preferences, while at the same time acknowledging the need to take addiction problems seriously, addiction treatment may move forward.

### Take Home Message

Addiction is a psychiatric condition. Putting addiction, whether in the context of an individual patient or in society at large, should be with the aim of reducing the negative consequences of substance use, and improving the quality of life.

## Questions That This Paper Raises

1. What is the most efficient way of reducing the social costs of alcohol and drug use: through policy aiming at reducing alcohol and drug use, or through policy targeting the indirect consequences, such as violence, accidents and social marginalization?
2. What is the relative contribution of “gateway drugs”, social risk factors and genetic risk in producing addiction?
3. How does the concept of addiction influence practice in the fields of prevention and treatment?
4. What is the worldwide influence of drug policy on human rights, democracy, and the living conditions of people, especially the poor?
5. Can harm reduction policy be integrated with ambitious treatment programmes?

## About the Author

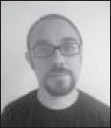



**Morten Hesse** graduated from Copenhagen University 1997 as an MSc of Psychology. Worked in inpatient and outpatient treatment in the greater Copenhagen area, and provides training in motivational interviewing, assessment of personality disorder and cognitive behavioural therapy in the addictions to professionals responsible for treatment of substance abuse in Denmark. Is the author or co-author of several articles in professional journals, and has served as a peer-reviewer for Addictive Behaviors.

## Publications (publications in Scandinavian languages omitted):

ReferencesHesseMAchieving abstinence by treating depression in the presence of substance-use disordersAddictive Behaviors2004296113711411523681410.1016/j.addbeh.2004.03.006HesseMPersonality disorders in substance abusers: validation of the DIP-Q through principal components factor analysis and canonical correlation analysisBMC Psychiatry20055251591068810.1186/1471-244X-5-24PMC1180835HesseMSocial workers’ ratings of comorbid personality disorders in substance abusersAddictive Behaviors200530/6124112461592513310.1016/j.addbeh.2004.12.002HesseMThe Readiness Ruler as a measure of readiness to change poly-drug use in drug abusersHarm Reduction Journal20063331643620810.1186/1477-7517-3-3PMC1395301
